# Oral and craniofacial research in the Generation R study: an executive summary

**DOI:** 10.1007/s00784-023-05076-1

**Published:** 2023-06-10

**Authors:** Agatha W. van Meijeren-van Lunteren, Xianjing Liu, Francien C. H. Veenman, Olja Grgic, Brunilda Dhamo, Justin T. van der Tas, Vid Prijatelj, Gennady V. Roshchupkin, Fernando Rivadeneira, Eppo B. Wolvius, Lea Kragt

**Affiliations:** 1 Department of Oral & Maxillofacial Surgery, Special Dental Care and Orthodontics, Erasmus University Medical Centre, PO Box 2040, 3000 CA Rotterdam, the Netherlands; 2 The Generation R Study Group, Erasmus University Medical Centre, PO Box 2040, 3000 CA Rotterdam, the Netherlands; 3 Department of Radiology and Nuclear Medicine, Erasmus University Medical Centre, PO Box 2040, 3000 CA Rotterdam, the Netherlands; 4 Department of Epidemiology, Erasmus University Medical Centre, PO Box 2040, 3000 CA Rotterdam, the Netherlands

**Keywords:** Epidemiology, Cohort studies, Dental health, Dental public health, Population-based

## Abstract

**Objectives:**

Oral conditions are of high prevalence and chronic character within the general population. Identifying the risk factors and determinants of oral disease is important, not only to reduce the burden of oral diseases, but also to improve (equal access to) oral health care systems, and to develop effective oral health promotion programs. Longitudinal population-based (birth-)cohort studies are very suitable to study risk factors on common oral diseases and have the potential to emphasize the importance of a healthy start for oral health. In this paper, we provide an overview of the comprehensive oral and craniofacial dataset that has been collected in the Generation R study: a population-based prospective birth cohort in the Netherlands that was designed to identify causes of health from fetal life until adulthood.

**Methods:**

Within the multidisciplinary context of the Generation R study, oral and craniofacial data has been collected from the age of 3 years onwards, and continued at the age of six, nine, and thirteen. Data collection is continuing in 17-year-old participants.

**Research outcomes:**

In total, the cohort population comprised 9749 children at birth, and 7405 eligible participants at the age of seventeen. Based on questionnaires, the dataset contains information on oral hygiene, dental visits, oral habits, oral health–related quality of life, orthodontic treatment, and obstructive sleep apnea. Based on direct measurements, the dataset contains information on dental caries, developmental defects of enamel, objective orthodontic treatment need, dental development, craniofacial characteristics, mandibular cortical thickness, and 3D facial measurements.

**Conclusions:**

Several research lines have been set up using the oral and craniofacial data linked with the extensive data collection that exists within the Generation R study.

**Clinical relevance:**

Being embedded in a multidisciplinary and longitudinal birth cohort study allows researchers to study several determinants of oral and craniofacial health, and to provide answers and insight into unknown etiologies and oral health problems in the general population.

## Introduction

Oral and craniofacial health plays a crucial role in the functioning of the human body and is an important contributor to general health and well-being. It was estimated in 2015 that 48% of the population globally suffered from one or more oral conditions [1]. Similarly, craniofacial anomalies are one of the most common congenital birth defects [2]. Therefore, the World Health Organization (WHO) emphasized the need for research on risk factors for oral and craniofacial diseases, and to develop effective (preventive) strategies in order to reduce the burden of those diseases [3]. Several social, behavioral, genetic, and environmental factors have already been identified as important contributors to the development of oral and craniofacial conditions [4], and conditions may be interrelated. However, prior studies were challenged by the complex (and high-dimensional) nature of oral and craniofacial disorders. The chronic and congenital nature and dynamic process of most oral and craniofacial conditions require longitudinal observations in a multidisciplinary setting. While it is not possible to study specific craniofacial defects in a healthy population-based birth cohort, it is possible to understand more about the etiology by studying subclinical (endo)phenotypes. Therefore, population-based cohort studies are essential to study and determine potential risk factors for oral and craniofacial conditions over time. Especially when making use of extensive measures and techniques to collect information on oral and craniofacial outcomes in an integrative setting including additional general health outcome measures and determinants.

Several cohort studies have measured oral health outcomes in the past. Recently, Peres et al. [5] presented an overview of existing oral health–related birth cohort studies worldwide. Of the 120 existing oral health–related birth cohort studies, there are 48 prospective cohort studies. The majority of these cohort studies are from high-income countries, and the USA, Australia, Brazil, and Sweden are the most representative. Dental caries is the most frequently investigated oral condition, followed by simple determination of oral microbiota and the level of dental plaque. However, there is a limited number of longitudinal cohort studies that follow children for a longer period until adulthood performing comprehensive assessments. Furthermore, only a few cohort studies have included oral and craniofacial assessments based on radiographs [6, 7], or facial three-dimensional (3D) images [8, 10]. Lastly, merely a few (small) longitudinal studies have collected dental plaque samples to determine dental biofilm composition extensively [11, 14]. To our knowledge, the most comprehensive oral and craniofacial data collection to date is performed by the Generation R study: a population-based prospective multiethnic birth cohort started in 2002 and follows children of almost 10,000 women from fetal life onwards [15].

The aim of this research program is to improve oral and craniofacial health through research and its translation to palpable health applications. We accomplish our mission by implementing technological advances in an observational epidemiological setting like the Generation R study. In this paper, we aim to provide an overview of all the oral and craniofacial outcomes measured in the Generation R study from birth until puberty onwards, to provide insight into the current and future research endeavors and research collaborations arising from this resource, enriched by its multiethnic background and longitudinal design.

## Methods

### Cohort design

Details about the enrolment procedure, data collection, and study population have been described previously in detail [15, 17]. In short, the Generation R study is a population-based prospective birth cohort study conducted in Rotterdam, the Netherlands. Women with a due date between April 2002 and January 2006 and who were registered inhabitants in the municipality of Rotterdam at the time of delivery were eligible to participate in the Generation R study.

The general design, research aims, and specific measurements performed in the Generation R study have all been approved by the Medical Ethical Committee of Erasmus MC, University Medical Center Rotterdam. New measurements are only introduced into the study after approval of the Medical Ethical Committee. Participants need to give written informed consent for each phase of the study (fetal, preschool, childhood, and adolescence period). From the age of 12 years onwards, children must sign their own consent form, in accordance with Dutch Law. At the start of each phase, children and their parents receive written and oral information about the study. Even with consent, when the child or the parents are not willing to participate actively, specific measurements are skipped or no measurements are performed at all.

The complete Generation R study has been approved by the Medical Ethical Committee of the Erasmus MC and conducted according to the World Medical Association Declaration of Helsinki. All examinations, interviews, and questionnaires were carried out after approval of the ethical committee, and after obtaining written informed consent from participants or their parent(s) or legal guardian(s). For each phase and component of the study program, new approval from the ethical committee was given, and informed consent forms were signed by participants. The reference numbers of the ethical documents per phase are as follows: phase 1 (fetal period) MEC 198.782.2001.31; phase 2 (0–4 years) MEC 217.595/2002/202; phase 3 (5–8 years) MEC-2007-413; phase 4 MEC-2012-165; MEC 2015-749 NL55105.078.15; MEC 2015-749, NL5105.078.15). The ethical approval documents and blank consent forms are available upon request.

Measurements started during early pregnancy and continued in early life. The prenatally recruited cohort is followed until young adulthood. During the whole study, extensive assessments have been performed on mothers, fathers, and children. The study group is a multiethnic cohort, representing a diversity of ethnic groups including Western (Dutch, European, North-American, Indonesian, Japanese) and non-Western participants (Surinamese, Moroccan, Turkish, Cape Verdean, African, Asian, South and Central American) [18, 19]. Besides, it includes participants from different socioeconomic backgrounds indicated, for example, by parental educational level, household income, parental employment status, and receiving financial benefits.

### Measurements and data collection

From the age of 3 years, the first oral health data were collected through parental questionnaires. Additionally, from the age of six onwards, oral health data were collected through researcher-performed measurements, based on protocols specifically outlined for this purpose. Currently, the data collection is ongoing in 17-year-old participants. In Table 1, an overview of all performed and ongoing measurements is presented. The measurements form the basis of the oral health data collection within the Generation R study. Besides the ongoing measurements on several oral and craniofacial outcomes, scoring data based on existing raw data is still performed and planned. Based on the available data within the Generation R study, several research projects are currently ongoing where oral or craniofacial outcomes are linked with potential determinants or general health outcomes. Moreover, the follow-up of the participants until at least young adulthood will generate longitudinal information on oral health enabling oral and craniofacial health research in a dynamic life-course framework. A description of the data obtained based on measurements will follow.

**Table 1:** Overview of data collection and measurements within Generation R

Measurements	Time point(s):				
	3 years	6 years	9 years	13 years	17 years (ongoing)
Parental questionnaires					
Toothache and difficulties eating	x				
Caries diagnosed by dentist	x				
Tooth brushing frequency	x	x	x	x	x
First age dental visit and reason		x			
Dental visit		x	x	x	x
Oral health–related quality of life			x	x	
Subjective orthodontic treatment need			x	x	
Suspected obstructive sleep apnea			x		
Type of orthodontic treatment				x	
Subjective result of orthodontic treatment				x	
Permanent teeth extraction for orthodontic treatment				x	
Abnormal oral or mouth habits				x	x
Visit oral hygienist				x	x
Oral hygiene methods				x	x
Maternal dental care insurance					x
Removal third molars					x
Mobile application for children					
Oral health–related quality of life				x	x
Researcher-performed measurements					
Intra-oral photographs		x		x	x
Frontal and buccal dental photographs			x		
3D facial images			x	x	x
Orthopantomogram and lateral cephalogram			x	x	x
Dental biofilm sample				x	x

#### Questionnaire data

At the age of 3 years, the first questionnaire including dental health–related questions was sent to the home addresses of participating children to be filled out by the parents or caregivers. At first, the questions regarded tooth brushing frequency, toothache, and dentist-diagnosed caries only. From the age of 6 years onwards, the oral health questions were supplemented with questions about (reasons for) dental visits. From the age of nine onwards, additional questions concerning subjective orthodontic treatment need and obstructive sleep apnea were added. At the age of thirteen, questions regarding orthodontic treatment, oral hygiene, and abnormal oral habits were included in the questionnaire. At the age of seventeen, participating young adults are directly addressed to answer the oral health questions partially via postal questionnaires.

Questionnaires that were sent and returned were scanned and manually entered into an electronic database. Multiple-choice questions were transformed into categorical response variables, and open text fields were cleaned and recoded to categorical response variables. Further information on the logistics and data processing of the questionnaires has been described earlier [15].

A mobile application was developed to obtain information on oral health behavior. The application and corresponding privacy and data storage were kept under control by the departments of Communication, Security, Legal Affairs, and the Data Protection Officer of the Erasmus Medical Center.

The oral health part within the app consists of repeated questions regarding perceived oral health status and last dental visits. At the age of thirteen, the application was used in a selection of participants from the Generation R study population for validation purposes. At the age of seventeen, data collection via the mobile application is continued, and all eligible participants will be invited to install and use the mobile application.

#### OHRQoL

At the age of nine and thirteen, oral health–related quality of life (OHRQoL) was assessed through a validated Child Oral Health Impact Profile (COHIP) short form. The COHIP short form measures the OHRQoL of the child with eleven questions, covering the different domains of oral health, including social-emotional well-being, functional well-being, and school and peer interaction [20]. At the age of seventeen, the OHRQoL questionnaire was administered via the Generation R mobile application (described in detail above). The questions were answered on a 5-point Likert scale and finally summed for each individual. The total score ranges from 0 to 55 with higher scores indicating better OHRQoL.

#### Dental caries

At the age of six, intra-oral photographs were made using an intra-oral camera (Poscam USB intra-oral autofocus camera, Digital Leader PointNix, 640 × 480 pixels). Photos were taken after tooth brushing and the removal of excess saliva. At the age of nine, oral photographs of the dentition were taken from three perspectives: frontal view, left buccal view, and right buccal view using a camera Panasonic Lumix DMC-TZ7. At the age of thirteen, oral photographs were taken using a quantitative light fluorescence (QLF) camera (Qraycam™ Pro (Inspektor Research Systems BV)). The whole dentition was captured in at least five white- and blue-light pictures using cheek retractors and after children had brushed their teeth. The same method applies to the ongoing data collection of intra-oral photographs at the age of seventeen.

Intra-oral photographs at the age of six were scored for dental caries by one single calibrated dentist. Ten percent of the photographs were scored by a second dentist. Intra-rater reliability (kappa = 0.80) and inter-rater reliability (kappa = 0.76) were evaluated for caries on a subsample of 10% [21]. The use of intra-oral photographs for scoring dental caries in epidemiological studies showed high sensitivity (85.5%) and specificity (83.6%) compared to the oral examination in a previous validation study [21]. Dental caries was assessed in the primary dentition using the decayed, missing, and filled teeth (dmft) index [21, 22]. At the age of thirteen, the intra-oral photographs were scored for dental caries by two calibrated researchers. Intra-rater reliability (weighted kappa = 0.94) and inter-rater reliability (weighted kappa = 0.84) were evaluated based on a subset of 100 participants. The use of the QLF camera for scoring dental caries in epidemiological studies showed high sensitivity and specificity compared to the clinical visual tactile inspection in a previous validation study [23]. Dental caries was assessed in the permanent dentition using the decayed, missing, and filled teeth (DMFT) index [22]. Teeth were scored as decayed when enamel breakdown was visible, using International Caries Detection and Assessment System (ICDAS) score three as a cut-off [24], which could be observed by white spot lesions and brown carious discoloration. Missing teeth were scored when elements were likely missing due to caries, verified on panoramic radiographs from the age of nine where fillings or severe caries could be detected. Fillings were scored when teeth were restored due to caries. Sealants and restorations due to trauma were not taken into account for the total DMFT score.

#### Developmental defect of enamel

Developmental defects of enamel in the deciduous and permanent dentition were scored using the (European Academy of Paediatric Dentistry) EAPD criteria for the assessment of Deciduous Molar Hypomineralisation (DMH) and Molar Incisor Hypomineralisation (MIH) using intra-oral photographs at the age of six (as described in the section on dental caries) [21, 25]. Ten percent of the photographs were scored by the same and a second dentist using the same method, to evaluate intra-rater-reliability (kappa = 0.95) and inter-rater reliability (kappa = 0.62) [21]. The use of intra-oral photographs for scoring DMH in epidemiological studies showed high sensitivity (72.3%) and specificity (92.8%) compared to the clinical visual tactile inspection in a previous validation study [21].

#### Orthodontic treatment need

Orthodontic treatment need at the age of nine was assessed using frontal and buccal photographs, 3D images, and panoramic radiographs. The measurements are described in other paragraphs of this manuscript; see “Dental caries,” “Dental development,” and “Facial shape.” Determination of orthodontic treatment need was done by one trained researcher and based on the Index of Orthodontic Treatment Need (IOTN) criteria, and consisted of a dental health component (DHC) and aesthetic component (AC) [26]. Assessment of the IOTN on a combination of photographic and panoramic radiographic records has been validated previously [27]. Intra-rater reliability (linear weighted kappa = 0.84) and inter-rater reliability (linear weighted kappa = 0.68) were calculated and showed good agreement [27].

#### Dental development

At the age of nine and thirteen, dental panoramic radiographs and lateral cephalograms were taken in a standardized manner using a digital dental imaging unit (OP/OC 200D; Tuusula, Finland). Glasses, jewelry, or any other metal objects were removed before the measurements were taken using a stationary and uniform positioning. The machine settings for the panoramic radiographs were as follows; voltage and current: 66 kV and 6.2 mA; exposure time: around 14 s; source-image distance (SID): 487 mm; and dose area product (DAP) reference levels between 40 and 66 mGy/cm^2^. For the panoramic radiographs, participants were instructed to stand as straight as possible, take a grip on the handles, and place the bite fork in the mouth. After the research assistant positioned the participants with the use of the positioning lights and the moving head and temple supports, participants were asked to take a small step forward, to swallow, and to leave the tongue in the upper palate while the radiograph images are taken. The machine settings for the lateral cephalograms were as follows; voltage and current: 85 kV and 13 mA; exposure time: around 7 s; SID: 1745 mm; and DAP reference levels between 3 and 5 mGy/cm^2^. For the lateral cephalogram, participants were instructed to stand under the cehpalostat. The research assistant adjusted the cephalostat to the proper height and introduced the ear rods; also, the nose support is adjusted by hand. Participants were asked to close their lips while the radiograph was taken. At the age of seventeen, the same method is applied.

Panoramic radiographs were used to ascertain dental development at both the age of nine and thirteen using the Demirjian method [28]. According to the Demirjian method, 7 teeth excluding third molars located on the left side of the mandible were scored with 1 of the 8 developmental stages (A–H) depending on the calcification of the crown and root. Following this approach, the calculation of dental age is derived from the developmental stages of the teeth present in the lower-left quadrant. One experienced examiner (BD) determined the eight stages of development (1–8) for all permanent teeth located in the lower-left quadrant (excluding the third molar). If the permanent tooth in the left mandible was congenitally missing, the stage of development was assessed from the corresponding tooth on the right side. The obtained stages of development were weighted for boys and girls using the Dutch, French-Canadian, Belgian, and International Demirjian dental age standards [28, 30]. From each dental age standard, a dental maturity score was calculated by summing the weighted scores of the obtained developmental stages of the 7 lower mandibular teeth, consequently. Lastly, tables from respective dental age standards were used to convert the summed dental maturity score to dental age for boys and girls [28, 30]. Dental age calculated by the Dutch standard consistently presented the best approximation with chronological age in our study population; hence, it was used as a proxy of dental development in the subsequent statistical analysis. A second examiner scored a subsample of 100 subjects to assess inter-rater agreement (ICC = 0.65 to 0.80) [31]. Hypodontia was also assessed at the age of nine. Based on a subset of 20 panoramic radiographs, inter-rater reliability was determined (ICC = 0.79–0.94) [32]. In addition, at the age of thirteen, information about third molar development, third molar agenesis, tooth extraction, dental trauma, root-canal treated teeth, and incidental findings is collected from the panoramic radiographs.

#### Craniofacial growth

At the age of nine, craniofacial growth was determined using lateral cephalograms (described in the section on dental development). Twenty-two cephalometric landmarks were used and from these points, 35 cephalometric parameters were derived: 16 angular, 15 linear, and 4 indexes. Details about the landmarks and parameters could be found elsewhere [33]. Cephalometric points were digitized by a trained investigator using Viewbox software, version 4.0 (dHAL Software, Kifissia, Greece). A second examiner scored a subsample of 93 subjects. Accordingly, the inter-observer agreement could be determined (ICC = 0.71 to 0.93), which showed good agreement [33]. Facial divergence, bi-maxillary growth, sagittal jaw relationship, ramus height, lower anterior facial height, and cranial base angle were identified as representing six skeletal craniofacial patterns using principal component analysis. Lip position, incisor angulation, and overjet were identified as representing three distinguished dental craniofacial patterns [34].

#### Mandibular cortical thickness

At the age of thirteen, mandibular cortical thickness was evaluated on dental panoramic radiographs using mental index (MI) and panoramic mandibular index (PMI). Mandibular bone mineral density has been associated with many factors such as extraction history, number of teeth present, denture use, and shape and thickness of the mandibular cortex [35, 37]. Also, studies have shown a positive correlation between mandibular bone mineral density, mandibular cortical thickness, and bone mineral density at other sites (e.g., femoral neck and lumbar spine) in the adult population [38, 39]. On top of that, the assessment of mandibular cortical thickness, via dental panoramic radiographs, has been suggested as a useful tool in the early diagnosis of osteoporosis in adults [40]. Therefore, we aimed to determine mandibular cortical thickness as a biomarker that can be used in early life assessment of bone quality. Details on the panoramic radiographs are described in the section “Dental development.” All the measurements were performed by a trained dentist. The MI was determined as mandibular cortical thickness measured on an axis that passes through the mental foramen and is perpendicular to a tangential axis drawn along the lower mandibular rim below the mental foramen. PMI was calculated as the ratio between MI and the distance between the lower border of the mandible and the upper (PMI superior) or lower (PMI inferior) border of the mental foramen (measured on the same vertical axis). Both indices were assessed bilaterally. A subset of 150 panoramic radiographs was reassessed by the same rater and independently trained dentist to calculate intra-rater reliability. Since indices were unable to be determined in a subset of panoramic radiographs, a deep learning technique involving transfer learning was utilized to establish them in that subset [41], a similar methodology was used earlier by Lee and colleagues in 2020 [42].

#### Facial shape development

At the age of nine and thirteen, three-dimensional (3D) facial images were taken of the children using a 3dMDface system (3dMD Inc., Atlanta, GA, USA) photogrammetric device by trained photographers. The system consisted of one modular camera unit in the middle and two modular camera units at the side, and it was calibrated on a daily basis. Image acquisition took place in a designated 3D imaging room with no windows and a consistent amount of ambient light. An adjustable chair was used in a fixed position, to assure a standard level of height and fixed distance between subjects and the camera system. Participants were asked to remove glasses and to wear a surgical hat that prevents the hair from covering the forehead or ears. During image capturing, all participants faced towards the middle modular camera unit and had a neutral facial expression with the eyes open. For each child at the age of nine and thirteen, two types of images were collected: dental (with lips open to show all teeth) and non-dental (with lips closed). At the age of seventeen, only non-dental 3D images are taken. Non-dental 3D facial images of mothers were taken at the child’s age of nine only, using the same procedure as the child.

Based on the raw 3D image data, a 3D facial shape dataset was built following the large-scale facial model (LSFM) pipeline [43]. Each 3D facial shape is modelled using Graph [44]. Then, a 3D auto-encoder [45], which consists of an Encoder and a Decoder, was used to tackle the high-dimensional facial shape data and generate the facial endophenotypes. Specifically, the Encoder compresses the high-dimensional facial shape into $\mathrm{N}$ latent features as low-dimensional representations, while the Decoder reconstructs the 3D facial shape from the latent features. By minimizing the error between the input and reconstructed facial shape, the main morphology of faces is captured in the $\mathrm{N}$ latent features which we defined as facial endophenotypes. In order to make a trade-off between reconstruction error and dimensional complexity, we conducted experiments on different numbers of facial endophenotypes, and the optimum number was found to be 200. Therefore, each 3D facial shape is finally represented by 200 facial endophenotypes: when the value of one endophenotype changes (larger or smaller), there will be a corresponding deformation in the facial shape. These facial endophenotypes could be used for general epidemiological or clinical research projects, where the association between an exposure and each of the facial endophenotypes can be mapped back to the facial shape via the Decoder.

#### Oral microbiome

At the age of thirteen, plaque samples were collected by rubbing a cotton swab over the buccal sides of the right lower permanent molars. In the case of orthodontic treatment that covered the buccal side of the teeth, the swab was taken from the lingual side of the right lower permanent molars. The cotton swab was placed in a dry tube and directly stored in a local fridge and the same day transported to central storage where it was stored in a freezer at − 80 °C. At the age of seventeen, the dental plaque samples were directly stored in a local freezer at − 20 °C and twice a week transported to a central storage where samples were stored at − 80 °C and processed for oral microbiome 16S profiling (described below).

DNA was isolated from the dental plaque swabs using the Stratec InviMag Universal kit (STRATEC Molecular, Berlin, Germany) on the KingFisher Flex (Thermo Fisher Scientific, Waltham, MA, USA). Amplicon sequencing of the DNA isolates targeted the V3-V4 hypervariable region of the 16S rRNA gene. Amplicons were formed by the primers described by Fadrosh et al. [46], and sequenced on the Illumina MiSeq platform (San Diego, CA, USA).

#### Incidental findings

Within the Generation R study, it is required to systematically check images and examinations of participants to observe any potential incidental findings. An incidental finding is an observation of a potential presence of a health problem or disease. All dental panoramic radiographs and lateral cephalograms were checked by a dentist and senior researcher to identify potential abnormalities such as hypodontia, third molar agenesis, supernumerary teeth, taurodontism, peg-shaped teeth, dental trauma, tooth abnormalities of position, pathological and non-pathological lesions of jaws, and craniofacial complex (for example, dense bone islands, cysts, or tumors). In consultation with the senior clinician (EBW), potential findings were discussed, and a decision was made to inform the parents of the participants and their dentist in order to take any action.

## Research outcomes

### Study sample

**Fig. 1:**
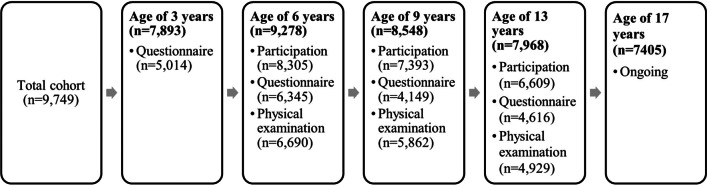
Flowchart indicating the timing and participation rates of oral and craniofacial measurements from birth until adolescence

In total, 9778 mothers were enrolled at the start of the study and gave birth to 9749 live-born children. At the age of 6 years, 8305 children (85%) participated in the study, at the age of 9 years, 7393 children (76%) participated in the study, and in the early adolescence period (13 years), 6609 children (70%) participated in the study (Fig. 1). For the next phase, 7405 eligible participants at the age of 17 years have been invited. This means we are in a position to perform a longitudinal analysis from pregnancy/birth with transitions from childhood to puberty and adulthood.

### Research lines

In the past years, several research areas within the Generation R study have been set up using the before-mentioned data linked with the extensive data collection within the Generation R study. In Table 2, an overview of the research output is given, and more details about the findings are provided hereafter.

**Table 2:** Overview of research output

OHRQoL
Dental caries [47]
Subjective orthodontic treatment need [48]
Socioeconomic indicators [49]
Self-esteem [50]
Ethnic background [51]
Objective orthodontic treatment need [52]
Dental caries
Anti-tissue transglutaminase autoimmunity (TG2A) [53]
Ethnic background [54]
Socioeconomic indicators [55]
Genetic determinants, consortium-based genome-wide meta-analysis [56]
Complicated pregnancy [57]
Breastfeeding [58]
Neighborhood characteristics [59]
Prenatal, perinatal, and early childhood vitamin D status [60]
Developmental enamel defects
Dental caries [61]
Relationship between hypomineralisation in deciduous and permanent dentition [62]
Maternal use of medicines during pregnancy [63]
Pre- and postnatal determinants (e.g., alcohol consumption, birth weight, fever episodes, ethnic background) [64]
Bone mineral content [65]
Anti-tissue transglutaminase autoimmunity (TG2A) [53]
Vitamin D concentrations during pregnancy, early and late postnatal period [66]
Dental development
Changes in dental maturity [67]
Hypodontia [32]
Maternal thyroid function [68]
Crowding and impaction [69]
Genome-wide association study on tooth agenesis [70]
Ancestry [31]
Vitamin D during pregnancy and early life [71]
Prenatal folate and vitamin B_12_ concentrations [72]
Craniofacial growth
Dental development [33]
Hypodontia [34]
Facial shape
Prenatal alcohol exposure [73]

#### Oral health–related quality of life (OHRQoL)

OHRQoL at the age of nine was related to subjective orthodontic treatment, and this relationship was not based on self-esteem [48, 50]. Progressively greater orthodontic treatment need has increasingly negative impacts on children’s OHRQOL [52]. Moreover, severe dental caries at the age of six were associated with a lower OHRQoL at the age of nine [47]. Also, children with a low family socioeconomic position had consistently lower OHRQoL [49] and children with a foreign ethnic background had a significantly lower OHRQoL than native Dutch children [51].

#### Dental caries

In a consortium-based genome-wide meta-analysis, few single variants were modestly associated with caries status [56]. Levels of anti-tissue transglutaminase autoimmunity (TG2A) were not associated with dental caries at the age of six [53]. No associations were found between several pregnancy complications and dental caries experience during childhood [57]. A weak association between serum 25(OH)D concentrations and risks of caries in primary teeth was found [60]. An association between prolonged breastfeeding and an increased risk of childhood dental caries was found [58]. Compared to native Dutch children, children with a Surinamese, Turkish, Moroccan, and Cape Verdean background had significantly higher odds for dental caries [54]. Furthermore, dental caries is more prevalent among 6-year-old children with a low socioeconomic status [55]. Lastly, it was found that childhood caries and the use of dental services differ between neighborhoods, and living in a deprived neighborhood is associated with increased dental caries and decreased yearly use of dental services [59].

#### Developmental defect of enamel

Deciduous molar hypomineralisation (DMH) and the country of birth of the mother play a role in the prevalence of dental caries [61]. A significant association was found between the occurrence of DMH and MIH (molar incisor hypomineralization), which suggests a shared etiology [62]. Furthermore, maternal use of antibiotics and allergy or asthma medication during pregnancy did not seem to play a role in the development of a child’s DMH [63]. Ethnic background, alcohol consumption during pregnancy, low birth weight, and fever in the first year of the child’s life were significantly related determinants of DMH [64]. Levels of anti-tissue transglutaminase autoimmunity (TG2A) were not associated with specific enamel defects at the age of six [53]. In addition, it was found that total body bone content is associated with DMH but not with MIH during childhood [65], and vitamin D concentrations in prenatal, early postnatal, and later postnatal life were not associated with the presence of DMH or with MIH at the age of six [66].

#### Dental development

In a collaborative study with the Nijmegen Growth study analyzing changes in dental maturity in Dutch children born between 1961 and 2004, dental maturity score was significantly and positively associated with the year of birth, gender, and age in Dutch children, indicating a trend in earlier dental development during the observation period from 1961 to 2004 [67]. Children with hypodontia have delayed dental development [32]. Also, in another study, accelerated dental development is associated with a lower occurrence of crowding, impaction, and hypodontia [69]. No association was found between maternal thyroid function during pregnancy and dental development of the child [68]. Based on a geographic and genetic perspective, differences in dental development exist in a population of heterogeneous ancestry [31]. In a consortium-based genome-wide meta-analysis, nine novel risk variants were associated with tooth agenesis [70]. In a study about maternal and neonatal 25(OH)D concentrations and dental development in childhood, lower vitamin D levels during mid-pregnancy or at birth were associated with higher dental age of children, and higher developmental stages of the mandibular teeth [71]. Maternal folic acid supplementation delays dental development of children by 1–2 months of dental age, whereas maternal folate and vitamin B_12_ concentrations in early pregnancy do not affect the timing of a child’s dental development [72].

#### Craniofacial growth

Children with mild hypodontia have distinctive skeletal and dental features [34]. The findings of another study show that dental development is associated with specific dental and skeletal cephalometric characteristics in school-age children [33].

#### Facial shape

The most important findings from a study that examined the association between prenatal alcohol exposure and children’s facial shape were that very low alcohol consumption during pregnancy (< 12 g absolute alcohol per week) is associated with children’s facial shape. This association attenuates as children grow up, and alcohol consumption before pregnancy showed a similar association [73].

## Discussion

This paper shows the complete overview of the oral and craniofacial data collection methods and data that has been collected so far within the Generation R cohort study. The Generation R study is a large population-based birth cohort studies collected data on multiple health (related) outcomes including oral and craniofacial conditions in children and young adolescents from birth to puberty. The integration of the oral and craniofacial data collection within the multidisciplinary Generation R cohort study enables the researchers to study oral and craniofacial diseases in an epidemiological manner linking it to a range of potential determinants including endocrine, environmental, genetic, epigenetic, microbiome, lifestyle, nutritional, infectious, and sociodemographic factors [15]. To the best of our knowledge, there is no other pediatric cohort with such an extensive data collection concerning oral and craniofacial measures. The description of its oral and craniofacial data in this paper (at least those with high prevalence) shows the importance of studying these outcomes in an epidemiological and life-course manner. The life-course approach has increasingly received attention in the last years, also in the field of oral and craniofacial health [74]. By enabling a better understanding of the development of oral and craniofacial conditions from birth onward, birth cohort studies form the basis for intervention strategies that aim to reduce the burden of oral health diseases within the population. Identifying determinants that contribute to the best possible start during the early years of life, in terms of oral and craniofacial health, may provide a stable basis for the oral health condition later in life.

Although being part of such a large cohort study, the logistics, time, and budget restrictions limit the use of expensive and extensive methods to collect data on oral health. The use of intra-oral photographs to determine dental caries and developmental defects of the enamel might underestimate the true prevalence of oral conditions compared to the visual examination with the use of bitewing radiographs [75]. Also, the determination of dmft and DMFT as a measure of dental caries in the primary and permanent dentition is not optimal using a longitudinal approach and does not allow us to study the severity of the disease throughout the years. Despite being unique by having collected panoramic radiographs longitudinally in a pediatric cohort, we cannot replicate our findings at the current moment as no other pediatric cohort have these or proxy measurements available. Also, the determination of gingival inflammation on dental photographs has not been validated further complicated by inconsistent light effects. Moreover, in order to identify both genetic and environmental causes for facial birth defects, craniofacial data at 6- or 12-month-old babies are needed. However, we did not collect 3D facial images at an age younger than 9 years. A nice addition to our data collection would be the collection of other oral samples such as subgingival plaque and saliva because it enables the analysis of different associations [76]. Saliva can also be used for hormone detection which is interesting in our adolescent population or to study the association with stress based on the cortisol levels in saliva. In addition, previous studies have collected oral microbiome samples from both mothers and children, which was not possible within our study setting. In general, the time limits, due to a strict schedule of all measurements, could decrease the precision of those measurements. The inferences drawn from the performed analyses depend on the precision of measurements. However, in large-scale population-based studies, the precision of measurements and the sample size must be weighed against each other. A major challenge, for example, is to prevent loss to follow-up, especially among the lower social class, which may lead to selection bias and thus influence the results of studies performed.

Given the large sample size, the oral and craniofacial data structure within the Generation R study is a unique resource and there are several possibilities for future research and collaborations. With the number of determinants that have been collected within this cohort, it could contribute to exposome research [77], which maps the exposures of an individual throughout life and how these are related to health. With the use of machine learning, the automatic determination of mandibular radiographic indices based on panoramic radiographs is currently being applied. This allows us to increase the sample size in exploring genetic determinants of mandibular cortical thickness, as an example of the plentiful machine learning applications. In future research, we will also focus on the epidemiology, genetics, and clinical implications of third molar agenesis and delayed dental development. Combined with extensive genetic and epidemiological measures, the presented 3D craniofacial dataset is valuable to unravel the complexity between genetics, facial morphology, and health outcomes. Specifically, to identify children at risk of developing cognitive problems related to brain anomalies, brain and facial shape will be mapped in relation to shared genetic or environmental factors. The same applies to the oral microbiome dataset, which constitutes the largest data set among children worldwide, especially in combination with the other extensive oral health measures that were taken at the same time points. The effect of several lifestyle factors, including nutrition, socioeconomic indicators, and health behaviors, will be investigated in relation to microbiome composition. In addition, when the data collection at the age of seventeen is finished, longitudinal sampling could be performed, enabling to study the development of oral microbiota over time. These measures could play an important contribution in the elucidation of the major oral health inequalities based on the ethnic or socioeconomic background that were found earlier. A future outlook of this project is to use oral microbiome knowledge in product development. Possibilities are to modulate the supragingival microbiota in different ways. For example, to stimulate the “healthy” microbiota via prebiotics and/or probiotics or to develop specific anti-bacterial treatments. Lastly, the introduction of the Generation R mobile application offers great opportunities to collect repetitively real-time data on several oral and craniofacial determinants directly from the participants.

There are currently also major efforts among national and international research institutions to collaborate or share oral and craniofacial health data [78, 81]. Collaboration with other research groups and cohort studies will lead to increased statistical power, using pooled analyses, but it could also improve the quality and impact of the research output. For example, the 3D facial dataset of children (Generation R) will be merged with a large dataset including the elderly (Rotterdam Study). With the merged dataset, a higher statistical power for a genome-wide association study (GWAS) could be reached and a geometric-deep-learning-based framework will be developed to predict the 3D facial shape from genotyped data. The datasets and their integration in a medical setting also offer great opportunities to collaborate with the clinic. For example, the healthy volunteers of the Generation R study could serve as control groups when comparing it to rare diseases such as cleft lip and cleft palate, but also ectodermal dysplasia. While the Generation R study data is not automatically publicly accessible due to legal and informed consent restrictions, the oral and craniofacial research group is open to collaboration and shared efforts. Requests for collaboration can be addressed to the secretary of the Generation R study (generation@erasmusmc.nl). These requests are discussed with the project team, and reasonable proposals will be presented within the Generation R Study Management Team concerning the aimed study question, overlap with ongoing projects, and logistic and financial consequences. The collaborative research project can be embedded in the research area of the oral and craniofacial research group after approval of the project by the Generation R Study Management Team and the Medical Ethical Committee of the Erasmus Medical Center.

Over time, results from the Generation R study could contribute to the development of strategies for optimizing oral and craniofacial health and healthcare for children. Generally, the research output of studies is limited to scientific journals and conferences. By using social media or the websites of our research groups (https://generationr.nl/researchers/ and https://oral-health.nl), important research output could reach a wider public. For example, the important research output of our study group gained publicity in national and local newspapers and television [54, 55], which was the basis to draw attention to the oral health inequalities that exist in the city of Rotterdam. This has led to important collaborations and initiatives to improve the oral health of children in primary schools in the city of Rotterdam. Besides, currently, a representative of the municipality of Rotterdam is part of the Generation R research and management team. This could enable collaboration and the initiation of relevant projects to improve the oral and craniofacial health status of children, and also to prevent the occurrence of related diseases in the population.

## Conclusion

This paper shows the importance and value of collecting comprehensive oral and craniofacial data in an epidemiological setting of a birth cohort study, like the Generation R study. Being embedded in a multidisciplinary population-based longitudinal study setting allows researchers to investigate the determinants of oral and craniofacial health, and to provide answers and insight to unknown etiologies and oral health problems in the general population. Using the life-course approach of a longitudinal cohort setting enables us to determine important factors that emphasize the contribution of a healthy start to sustainable oral and craniofacial health later in life.

## Data Availability

The datasets of this study are not openly available due to legal and informed consent restrictions. Reasonable requests to access the datasets should be directed to generation@erasmusmc.nl, in accordance with the local, national and European Union regulations.
